# An Innovative Approach for Management of Vertical Coronal Fracture in Molar: Case Report

**DOI:** 10.1155/2012/327812

**Published:** 2012-02-19

**Authors:** Ambica Kathuria, M. Kavitha, P. Ravishankar

**Affiliations:** ^1^Maulana Azad Institute of Dental Sciences, MAMC Complex, Bahadur Shah Zafar Marg, New Delhi 110002, India; ^2^Tamilnadu Goverment Dental College and Hospital, Near Chennai Fort, Chennai 600003, India; ^3^Thai Mooghambigai Dental College, Chennai, Tamilnadu, India

## Abstract

Unlike anterior teeth, acute exogenous trauma is an infrequent cause of posterior coronal vertical tooth fractures. Endodontic and restorative management of such fractures is a great challenge for the clinician. Newer advancements in adhesive techniques can provide successful intracoronal splinting of such teeth to reinforce the remaining tooth structure. This paper describes the diagnosis and management of a case of complicated vertical coronal fracture in mandibular first molar induced by a traffic accident.

## 1. Introduction

Coronal fractures of posterior teeth usually occur due to chronic masticatory trauma presence of large restorations or unrestored endodontically treated teeth [1, 2] and rarely due to acute trauma. If a visible separation occurs at the interface of segments along the line of fracture, such fractures are termed as “complete” as coined by Luebke [3]. The fracture commences in the crown and generally terminates near the cementoenamel junction or may extend apically into the root. Coronal fractures often involve a marginal ridge with the fracture extending in a mesiodistal direction. 

There is relatively scarce research on vertical tooth fractures, and several doubts still persist regarding their diagnosis and treatment. Management of such teeth should involve recognition of signs and symptoms and the provision of adequate restorations that protect the remaining tooth structure [4]. 

This paper attempts to highlight an innovative approach for the management of fractured fragments and preservation of remaining tooth structure in a case of posterior vertical coronal tooth fracture. Orthodontic wires and fiber-reinforced composites have been suggested as means for additional splinting of fractured segments.

## 2. Case history

**Figure 1:**
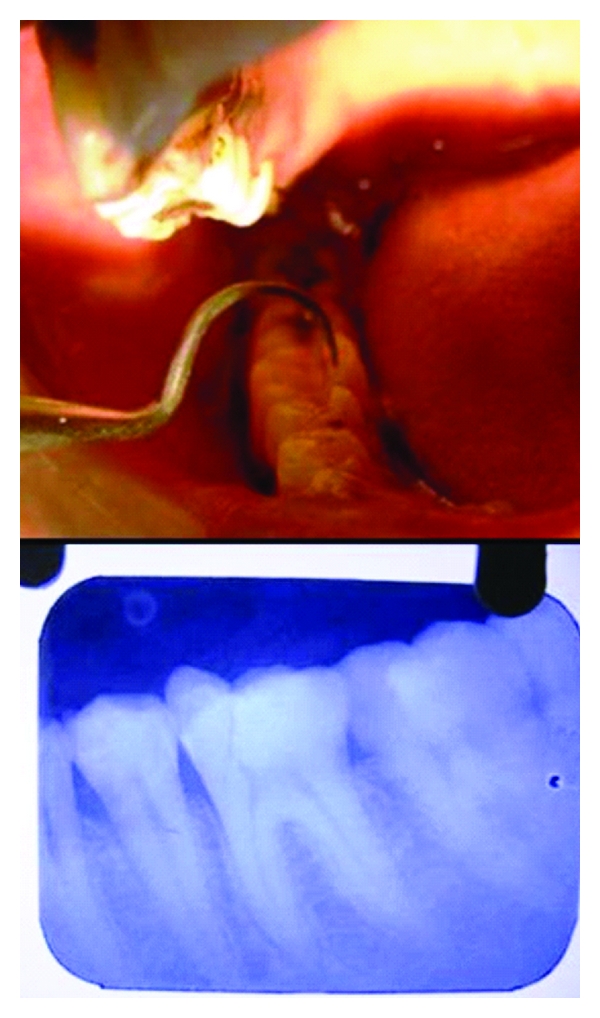
Vertical coronal fracture in mandibular molar, pre-operative radiograph.

A 35-year-old male patient presented with the complaint of pain in the lower right posterior tooth region for three days, after he met with a traffic accident. Examination revealed a vertical fracture in crown of right mandibular first molar with pulpal exposure (Figure 1). The fracture line was seen to extend mesiodistally more towards the mesiolingual aspect. Although the fragments were intact and undisplaced, wedging with a probe resulted in slight movement of the lingual segment indicating a complete fracture. All the tooth surfaces were carefully checked in dry field for presence of other cracks or craze lines. Gingiva around the tooth was palpated to check for possible evidence of an underlying dehiscence or fenestration to rule out vertical root fracture.

Radiographs were taken at different horizontal angulations. Fractured fragments were seen to be separated by a narrow radiolucent line extending to the CEJ (Figure 1). Hence the case was diagnosed as a vertical coronal fracture running mesiodistally into the dentin and pulp but confined to the crown of the tooth.

**Figure 2:**
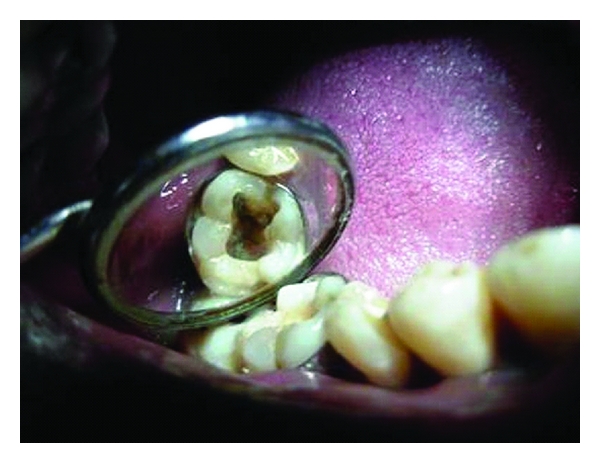
Splinting with orthodontic band.

**Figure 3:**
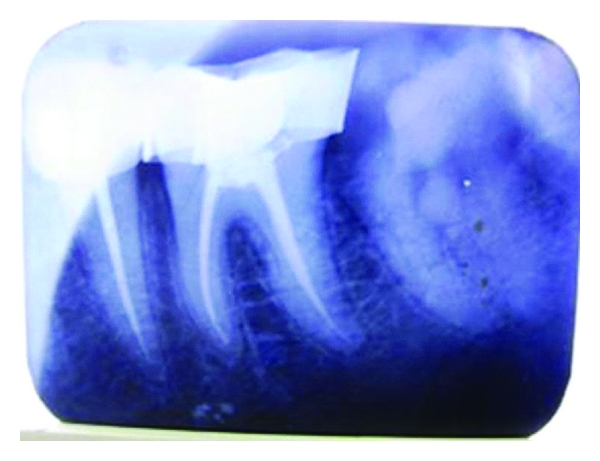
Postobturation radiograph.

The tooth was immediately adjusted out of occlusion. A preformed orthodontic stainless steel molar band (Ortho organizers, CA, USA) was cemented around the tooth to hold the fragments in position (Figure 2). This stabilisation also helped in rubber dam placement during the root canal treatment. Access opening was done under rubber dam isolation, pulp was extirpated, and temporary restoration was given. The patient was recalled after a week, and root canal therapy was completed (Figure 3).

**Figure 4:**
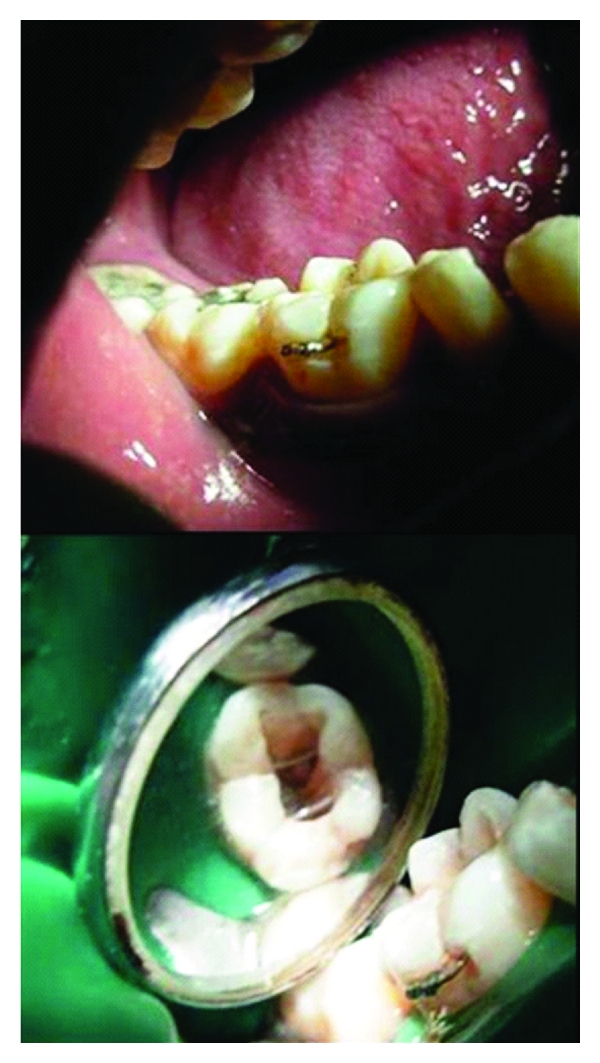
Wire reinforcement (buccal view), wire reinforcement (occlusal view).

On follow-up visit after 15 days, tooth was asymptomatic to chewing. Thereafter, through and through holes were created on buccal and lingual surfaces with very thin tapering fissure bur, and the band was gently removed. Slots 1 mm, deep were prepared on the buccal and lingual surfaces with fine straight fissure bur. An orthodontic stainless steel wire 26 gauge 0.012′′ (KC Smith) was passed through the holes in the crown buccolingually making a loop, and free ends of wire, were tied buccally in the prepared slot (Figure 4). This wire reinforcement acted as an additional splint for the fractured fragments. 

**Figure 5:**
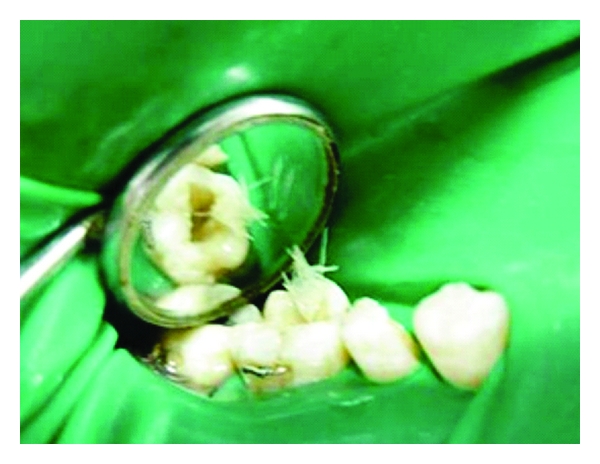
Intracoronal splinting with FRC.

**Figure 6:**
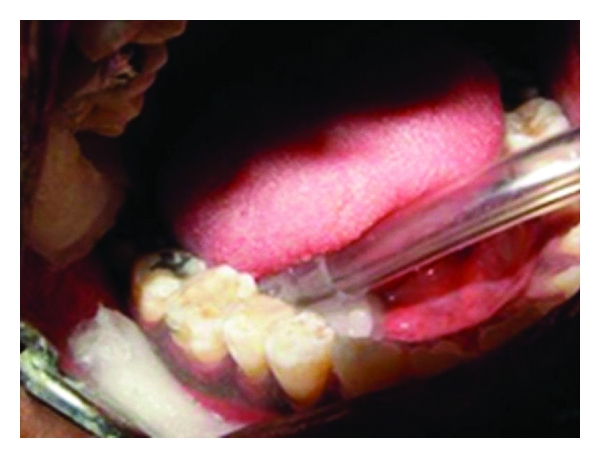
After light cure composite restoration.

After performing priming and bonding procedures, the cavity surfaces were coated with a layer of low-viscosity resin composite (Protect Liner F, Kuraray, Japan) within 1 mm of the preparation margins and kept uncured. A piece of leno weave ribbon formed of ultra-high -molecular-weight polyethylene fiber (UHMWPE) (Ribbond, Seattle, WA, USA) was cut and impregnated with adhesive resin (Figure 5). Excess material was removed with a hand instrument, and the fiber was embedded into the bed of uncured flowable resin from occlusal 1/3 of the buccal wall to occlusal 1/3 of the lingual wall. After curing for 20 s, the cavities were restored incrementally with composite and cured using a combination of pulse and progressive curing technique (Figure 6). Buccal and lingual slots were also restored with composite resin.

Follow-up was done three months later and then 6-month follow-up for three years. During follow-up appointments, clinical and radiographic examinations revealed no endodontic or periodontal problems, suggesting the efficacy of the treatment in retaining the fractured tooth.

## 3. Discussion

A posterior crown fracture due to acute trauma is an unusual type of dental injury [5]. Fractures that cross both marginal ridges usually involve pulp and may extend apically into the root. Salvage of such teeth definitely poses a challenge for the clinician. Hence newer conservative techniques are needed to address this situation. 

Coronal tooth fracture increases cuspal flexure under occlusal load and weakens the tooth. This decreases the stiffness of the tooth. Hence, a temporary restoration should protect a tooth from further deterioration during endodontic treatment. Pane et al. [6] proved that stainless steel bands reduce cuspal flexure by one-half compared to teeth without bands and, furthermore, doubled the fracture strength. The stainless steel band provides a good immediate treatment option to protect fractured teeth during root canal therapy. This paper suggests the use of stainless steel bands along with an additional splinting using stainless steel orthodontic wire. 

It is of outstanding importance to stabilize fractured teeth further weakened due to access cavity preparation [7]. Postendodontic restoration is a critical final step of successful endodontic therapy in these cases because the presence of fracture lines or cracks sometimes may even cause failure of the root canal treatment [8]. Thus, the preservation and reinforcement of the remaining tooth structure are important for the longevity of the treatment. Although cast restoration or cuspal coverage traditionally has been suggested as the final restoration, various studies have emphasised intracoronal strengthening of teeth to protect them against fracture [9]. Bonded restorations, especially fiber-reinforced composites (FRC), have been proposed for internal splinting of such teeth. It is documented that the fibrous assemblies can increase the effective fracture strength of the teeth. The fibers act as stiff bands when stretched over prefractured surfaces resisting crack opening and create a strong bridge between the fractured fragments [8].

It is recommended that the use of UHMW polyethylene in the form of a leno weave before restoring teeth with resin composite would provide an increase in fracture strength. This is explained based on the combined effect of the fiber modulus and the interwoven structure (which has fibers oriented in multiple directions), which allows for the forces to be distributed over a wider area, thereby decreasing stress levels. The fibers provide multiple stress paths for redistribution of imposed stresses to intact portions of the teeth, and away from the bonded surfaces [8]. 

It has been demonstrated that a pulse-curing technique can reduce stress development at the cavosurface margins, which avoids the formation of microcracks [10] and results in an improved marginal adaptation while maintaining excellent physical properties of composite resin [11].

A combination of incremental placement of composite resin and UHMWPE fiber reinforcement system is considered of paramount importance to further reduce polymerization shrinkage, reinforce the remaining tooth structure, and reduce the total composite volume [12].

This paper presents a useful clinical technique for vertical coronal fracture management. Though orthodontic wires have been used for extracoronal and intracoronal splinting of mobile teeth, its use for fractured tooth management has not been reported yet. Although wire reinforcement provides an economical alternative, its effectiveness in long run needs to be substantiated through further case studies.
